# Micro-Photographs in Histology, Normal and Pathological

**Published:** 1876-08

**Authors:** 


Micro-Photographs in Histology, Normal and Pathological. By Carl Sieler, M.D., in conjunction with J. Gibbons Hunt and Joseph G. Richardson, M.D. Published monthly, by J. H. Coates & Co., 822 Chestnut street, Philadelphia Price $6 per annum.

Vol i. No. i. contains: Plate i., section of skin transversely through the hair bulbs; plate ii., epithelium of lower lip; plate iii., pavement epithelium from a Triton; plate iv., endothelium from diaphragm of a Guinae pig. Vol. i. No. ii. contains: plate v., elastic connective tissue; plate vi., scirrhus of mammary gland; plate vii., new elastic connective tissue from omentum of a cat; plate viii., connective tissue corpuscles from cornea of a frog.

Each number will contain four elegant plates of both normal and pathological specimens, and each plate will be accompanied by a short description of the specimen, making prominent the most noteworthy features. The publication is designed for physicians who have not the means at hand or the time to make investigations of this character; and to the student of microscopy they are particularly useful for comparison, as they, by their superior photography, make salient many points that would not be noticed by an unpracticed eye.

